# In-Brace versus Out-of-Brace Protocol for Radiographic Follow-Up of Patients with Idiopathic Scoliosis: A Retrospective Study

**DOI:** 10.3390/children9040465

**Published:** 2022-03-25

**Authors:** Charles M. M. Peeters, Arthur J. van Hasselt, Frits-Hein Wapstra, Paulus C. Jutte, Diederik H. R. Kempen, Christopher Faber

**Affiliations:** 1 Department of Orthopaedics, University Medical Center Groningen, University of Groningen, 9713 GZ Groningen, The Netherlands; c.m.m.peeters@umcg.nl (C.M.M.P.); a.j.van.hasselt@umcg.nl (A.J.v.H.); f.h.wapstra@umcg.nl (F.-H.W.); p.c.jutte@umcg.nl (P.C.J.); 2 Department of Orthopaedics, OLVG Hospital, 1061 AE Amsterdam, The Netherlands; d.h.r.kempen@olvg.nl

**Keywords:** scoliosis, brace therapy, in-brace radiographs, out-of-brace radiographs, curve progression rate

## Abstract

The purpose of this retrospective study was to compare two standardized protocols for radiological follow-up (in-brace versus out-of-brace radiographs) to study the rate of curve progression over time in surgically treated idiopathic scoliosis (IS) patients after failed brace treatment. In-brace radiographs have the advantage that proper fit of the brace and in-brace correction can be evaluated. However, detection of progression might theoretically be more difficult. Fifty-one IS patients that underwent surgical treatment after failed brace treatment were included. For 25 patients, follow-up radiographs were taken in-brace. For the other 26 patients, brace treatment was temporarily stopped before out-of-brace follow-up radiographs were taken. Both groups showed significant curve progression compared to baseline after a mean follow-up period of 3.4 years. The protocol with in-brace radiographs was noninferior regarding curve progression rate over time. The estimated monthly Cobb angle progression based on the mixed-effect model was 0.5 degrees in both groups. No interaction effect was found for time, and patients’ baseline Cobb angle (*p* = 0.98), and for time and patients’ initial in-brace correction (*p* = 0.32). The results of this study indicate that with both in-brace and out-of-brace protocols for radiographic follow-up, a similar rate of curve progression can be expected over time in IS patients with failed brace treatment.

## 1. Introduction

Idiopathic scoliosis (IS) is a common three-dimensional deformity of the spine involving a coronal major curve Cobb angle exceeding 10 degrees and spinal rotation [1]. The prevalence of IS is approximately 3% for children younger than 16 years old, of which ten percent have progressive spinal curves and require treatment [2,3]. Severe curves with a Cobb angle exceeding 45–50 degrees have a high risk of progression in adulthood and are therefore often treated surgically with posterior spinal instrumentation and fusion using pedicle screws [4,5,6,7].

The best proven nonsurgical treatment is rigorous bracing during a number of years of the adolescent growth spurt with the aim of maintaining the curve below 45 degrees. A randomized and preference cohort trial reported a treatment rate success of 72% after bracing, compared to 48% after observation [2]. The success rate of bracing was mainly associated with compliance as there was a significant positive association between hours of brace wear and rate of treatment success [2,8]. Therefore, early detection of curve progression during brace treatment is important for motivational reasons as the most important positive factor influencing brace compliance is the patient’s desire to avoid surgery and to prevent curve progression [9].

To detect curve progression during brace treatment, regular follow-up radiographs are usually made at 6-month intervals [3]. According to the SOSORT bracing protocol, these radiographs should be taken out-of-brace to examine the effectiveness of treatment (level V of evidence) [3]. On the contrary, follow-up with in-brace radiographs has the advantage of the proper fit of the brace and allows in-brace correction to be evaluated. However, it has been assumed that detection of progression might theoretically be more difficult when taking in-brace radiographs, since the curve is partially corrected.

To date, there have been no studies that have analyzed these two different radiographic follow-up strategies for the ability to detect progression and the rate of progression. Therefore, this study will compare two standardized protocols for follow-up radiographs (in-brace versus out-of-brace radiographs) from two different scoliosis centers for the ability to detect curve progression over time in idiopathic scoliosis patients with failure of brace treatment.

## 2. Materials and Methods

### 2.1. Study Design

This retrospective study was approved by the Medical Ethical Review Board (RR-number: 201900088) and conducted in two different tertiary care centers for scoliosis. Two standardized protocols for follow-up radiographs (in-brace versus out-of-brace radiographs) were compared. The in-brace group consisted of patients who underwent surgical treatment for idiopathic scoliosis in the first tertiary center after failed brace treatment. The standard protocol of this hospital was to take in-brace follow-up radiographs. The ability to detect curve progression over time on the in-brace radiographs was analyzed and subsequently compared to the out-of-brace group of surgically treated idiopathic scoliosis patients with failed brace treatment in the second hospital. The standard protocol of the second hospital was to take the first radiograph in the brace to evaluate the in-brace correction and all subsequent follow-up radiographs out-of-brace. Wearing of the brace was discontinued for a minimum of 12 h before the out-of-brace radiograph was taken. For the out-of-brace radiographs, patients were instructed to take their brace off during dinner, sleep without their brace, and return to the hospital the next morning without wearing the brace. Before taking the radiographs, the time of discontinuation of brace wear was checked.

### 2.2. Patients

Patients from both medical centers were included in this retrospective study according to the following inclusion criteria: they were diagnosed with idiopathic scoliosis below 50 degrees (i), and had undergone surgical treatment for scoliosis after failed brace treatment (ii), follow-up of the bracing period with radiographs lasted for at least 18 months (to be able to detect progression) (iii), and radiographs and patients data were available in the electronic patient records or archives (iv) (Table 1). Patients with non-idiopathic or non-progressive scoliosis and those who had undergone previous spinal surgery during bracing period were excluded. Scoliosis progression was defined as an increase in Cobb angle of ≥5 degrees during the bracing period [10]. The Boston brace was used for all patients in both centers, and the prescribed brace dosage was at least 20 h per day [11]. Radiographs in braces other than the Boston brace were excluded.

### 2.3. Method of Measurements

In the in-brace group, all in-brace radiographs during the bracing period in UMCG were used for analysis. Two independent observers (AH and CP) separately measured the Cobb angle of the major curve of the scoliosis deformity in standing anteroposterior view of each radiograph of the included patients. Data of the in-brace group are presented as the mean of both observers. In the out-of-brace group, the Cobb angles of the major curves on the index radiographs followed by the Cobb angles on all radiographs out-of-brace during the bracing period were collected from the well-organized archives of OLVG. Since follow-up intervals varied widely, measurements of the in-brace group were clustered in intervals of 6 ± 3 months, starting from the date of the first in-brace radiograph to the last. In the out-of-brace group, measurements were clustered in the same intervals, but starting from the date of the last radiograph before bracing to the last out-of-brace radiograph in the bracing period. When two radiographs fell within the same time interval, their mean Cobb angle was used. Reasons for varied follow-up intervals were adjustments for patients’ individual needs (first brace, growth spurt, atypical or progressive curve, poor compliance) [3]. The initial in-brace correction was only calculated for patients where the time frame between pre-brace measurement and first measurement in-brace did not exceed 6 months.

### 2.4. Statistical Analysis

Patient characteristics’ comparability was assessed using independent sample t-test for continuous variables and the chi-square test for categorical variables. Curve progression was calculated by subtracting patients’ Cobb angle at the first included in-brace or out-of-brace radiograph from the Cobb angle at the following six-monthly intervals. A one-sample *t*-test was used to test for differences between the degree of curve progression in each group at the end of the brace treatment and zero, which stands for no curve progression. An independent t-test was used to test for differences in curve progression between both groups. Analysis of curve progression measures over time was conducted with linear mixed models for repeated measures with restricted maximum likelihood estimation, with adjustment for baseline Cobb angle score and initial in-brace correction and time included as a linear term. Possible interaction effects for group and time, baseline Cobb angle score (patients’ Cobb angle at the first included radiograph in-brace or out-of-brace) and time, and initial in-brace correction and time were examined. To evaluate whether the ability to detect curve progression over time with the in-brace protocol was non-inferior compared to the out-of-brace protocol, a non-inferiority analysis was performed. Since the recognized measurement error in measuring Cobb angles is 5 degrees, a non-inferiority margin of 5 degrees was used for the yearly curve progression rate [3]. This results in a non-inferiority margin of 0.4 degrees in monthly progression rate, which will be presented in the results as outcome measure. The in-brace protocol is considered non-inferior when the 95% confidence interval (CI) of the monthly progression rate does not exceed the non-inferiority margin of 0.4 degrees. SPSS Statistics for Windows, version 23.0 (IBM Corp., Armonk, NY, USA), was used for statistical analysis. A *p*-value < 0.05 was considered to be statistically significant.

## 3. Results

### 3.1. Patient Characteristics

Twenty-five patients fulfilled the inclusion criteria for the in-brace group with in-brace follow-up radiographs (Table 2). The mean age at surgery was 15.0 years (SD = 1.6), and twenty-two patients (88%) were female. The mean pre-brace Cobb angle was 40 degrees, and the mean preoperative Cobb angle out-of-brace was 58 degrees. The mean duration of treatment with a Boston brace was 4.1 years.

The out-of-brace group consisted of 26 patients with failed brace treatment, who received out-of-brace follow-up radiographs. There were no significant differences in mean age at the start of Boston brace treatment, age at surgery, gender ratio, pre-brace Cobb angle, number of patients with pre-brace Lenke classification curve type 1, brace initiation before menarche ratio, study follow-up duration, duration of brace treatment, and preoperative Cobb angle out-of-brace between the two groups (Table 2) [12]. However, the percentage of initial in-brace correction was significantly larger in the out-of-brace group (37%) compared to the in-brace group (20%, *p* < 0.01).

### 3.2. Curve Progression of Scoliosis

**Figure 1:**
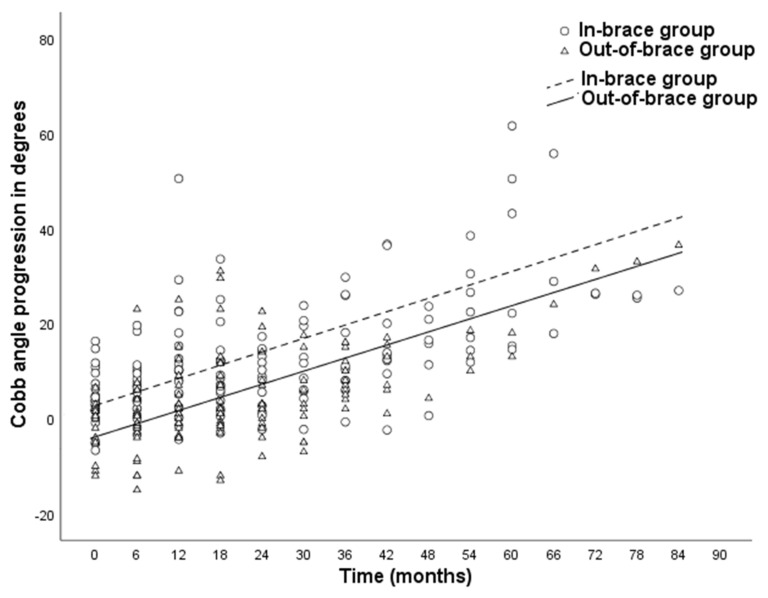
Cobb angle progression over time of the in-brace group and out-of-brace group. Cobb angle measurements on follow-up radiographs were clustered in time intervals of 6 months ± 3 months. The formulas for the estimated monthly Cobb angle progression in the in-brace (2.8 + 0.5*x) and out-of-brace group (−3.8 + 0.5*x) were formed using the mixed-effect model with adjustment for baseline Cobb angle score and initial in-brace correction and time included as a linear term.

Figure 1 presents Cobb angle progression over time of the in-brace and out-of-brace group. In both groups, significant curve progression was observed compared to baseline during the bracing period (*p* < 0.01). The mean curve progression at the end of the follow-up was 22.9 ± 15.3 degrees in the in-brace group versus 15.2 ± 7.9 degrees in the out-of-brace group (*p* = 0.03, see Table 3). Curve progression was only significantly higher in the in-brace group compared to the out-of-brace group at the first follow-up visit, with a mean difference of 6.6 degrees in Cobb angle. The mean difference of curve progression at the end of brace treatment was 7.6 degrees.

No significant differences in Cobb angle curve progression across time were established between the in-brace and out-of-brace group (*p* = 0.80). Additionally, no interaction effect was found for time and patients’ baseline Cobb angle (*p* = 0.98) or for time and patients’ initial in-brace correction (*p* = 0.32). The estimated monthly Cobb angle progression based on the mixed-effect model was 0.5 degrees in both the in-brace and out-of-brace group (Table 3). The criteria for non-inferiority were met, as the 95% CI did not exceed the predefined non-inferiority margin of 0.4 Cobb angle degrees. The mean study follow-up duration was 3.4 ± 2.0 years for the in-brace group and 3.3 ±1.3 years for the out-of-brace group (*p* = 0.78).

## 4. Discussion

In this study, two standardized protocols for follow-up radiographs (in-brace versus out-of-brace radiographs) from two different medical clinics were compared for the ability to detect clinically relevant curve progression over time in idiopathic scoliosis patients with failure of brace treatment. Curve progression was only significantly higher in the in-brace group compared to the out-of-brace group at the first follow-up visit, with a mean difference of 6.6 degrees in Cobb angle. This difference can be explained by the difference in baseline measurement, as the first radiograph in-brace was used as a reference for the in-brace group. In the out-of-brace protocol, the index radiograph just before the start of brace treatment was used as reference for future measurements. The radiograph that checks the correction and effectiveness of the brace cannot be used as a reference in the out-of-brace protocol. Since curves do not completely return to their original severity after temporary discontinuation of the brace, the out-of-brace group had a negative mean curve progression at the first follow-up visit. This explains the difference in progression at the start. After this first measurement, the rate of curve progression was not statistically significant any more between both groups. Consequently, this study shows that the protocol with in-brace radiographs was non-inferior regarding curve progression rate over time. However, switching between protocols results in a temporary inability to detect curve progression.

To our knowledge, there are no studies analyzing both in-brace and out-of-brace follow-up protocols for the ability to detect curve progression over time. The SOSORT bracing protocol recommends a quality check of the brace through an in-brace radiograph (level IV of evidence) and regularly performing out-of-brace radiographs to examine the effectiveness of bracing treatment (level V of evidence) [3]. In the literature, studies investigating curve progression in IS patients treated with brace therapy generally used out-of-brace radiographs at follow-up visits [10,13].

When interpreting the results of this study, a few limitations should be considered. This study was designed to determine and compare the rate of curve progression for both follow-up protocols. Therefore, only patients with curve progression were selected. This patient group was, however, considered as the most relevant for this study’s research question. Another limitation of this study is that the reason for failure of the brace treatment could not be investigated with the current study design. Furthermore, the results of this study were not based on an experimental study design but on retrospective observations. Although this study did not focus on predictive factors for curve progression, the patient characteristics were comparable between the in-brace and out-of-brace group, except for the initial-in-brace correction. In both groups, the mean initial in-brace correction was less than 45%, which is associated with brace treatment failure. Although in-brace correction has been described as an important predictive factor for brace failure, a minimum threshold has not been established. Previous studies have reported optimal cut-off values for initial in-brace correction varying between less than 10% and 45% as predictive of brace treatment failure [14,15]. In our study, 11.8% of the patients had an initial in-brace correction of less than 10%, whereas 84% of the patients had an initial in-brace correction of less than 45%. There was no interaction effect found for time and patients’ initial in-brace correction (*p* = 0.32). Therefore, the 18% difference in mean initial in-brace correction between both groups has probably not influenced the rate of curve progression. Other limitations are the relatively small patient groups and variation in follow-up intervals among included patients. No power analysis was performed. This was not considered as a problem for the interpretation of this study’s results, since the 95% confidence interval of the difference in monthly curve progression between the in-brace and out-of-brace group was very small (−0.09–0.12 degrees in Cobb angle) and within the non-inferiority margin of 0.4 degrees. So far, there are no evidence-based protocols, and current follow-up is based on an international consensus [3]. When signs of treatment failure were detected, physicians tended to deviate from this consensus to monitor patients more closely, which could explain the variation in follow-up intervals. A final limitation of this study is that a possible lack of compliance to the brace treatment was not monitored, which is an important factor for treatment failure.

The main therapeutic goal of bracing is to halt the scoliosis curves from progression and prevent the need for surgical treatment. During brace treatment, patients are regularly seen to check proper brace fit and verify its usefulness [3]. The early detection of curve progression could be important for motivational reasons to improve brace compliance. Often, out-of-brace protocols for radiologic follow-up include temporary discontinuation of the brace, as it allows visualization of progression above the curve size at the start of treatment. On the contrary, the major advantage of in-brace radiographic follow-up is that proper curve correction can be evaluated and brace corrections can be made if necessary. The theoretical drawback of in-brace follow-up radiographs is decreased detectability of curve progression due to partial correction of the curve by the brace. This study shows that the ability to detect curve progression was similar in two cohorts of patients with in-brace and out-of-brace radiologic follow-up protocols. Switching between protocols during the brace treatment would not be recommended, as this results in a period in which a physician is blinded to progression since the reference radiographs vary between protocols. However, when progression is demonstrated on subsequent follow-up radiographs and the major curve Cobb angle exceeds 40 degrees, a one-time switch from the protocol with in-brace radiographs to the protocol with out-of-brace radiographs should be considered. This is because curves exceeding 45–50 degrees are often treated surgically, and out-of-brace radiographs can provide more useful information for clinical decision making [4,5,6,7]. Although the protocol with in-brace radiographs was non-inferior in this study regarding curve progression rate over time, the severity of the major curve Cobb angle is still underestimated by in-brace radiographs. A potential delay in surgical treatment could occur, and therefore the out-of-brace protocol is preferred for potential surgery candidates. For non-potential surgery candidates, for example patients with a major scoliosis curve below 40 degrees, a clinician might consider using the protocol with in-brace radiographs in order to evaluate the curve correction at each follow-up visit so that brace corrections can be made if necessary.

## 5. Conclusions

In conclusion, this study shows that the rate of curve progression is similar in patients with failed brace treatment when checked with in-brace and out-of-brace radiologic follow-up protocols. For potential surgery candidates with larger major curve Cobb angles, the protocol with out-of-brace radiographs or a switch from protocol with in-brace radiographs to out-of-brace radiographs is, however, preferred in daily practice, since out-of-brace radiographs can provide more useful information for clinical decision making. For patients with a smaller scoliosis curve, the protocol with in-brace radiographs can be considered in order to evaluate the curve correction so that brace corrections can be made if necessary. 

## Figures and Tables

**Table 1:** Patient inclusion.

Inclusion Criteria	Exclusion Criteria
Diagnosed with idiopathic scoliosis	Diagnosed with non-idiopathic scoliosis
Major curve Cobb angle was <50 degrees at study inclusion	Patients with non-progressive scoliosis ^1^
Patients underwent surgical correction after failed brace treatment	Previous spinal surgery during bracing period
Follow-up of the bracing period was with radiographs	Radiographs in braces other than the Boston brace
Follow-up of the bracing period was at least 18 months	
Radiographs and patients data were available	

^1^ Scoliosis progression was defined as an increase in Cobb angle of ≥5 degrees during the bracing period.

**Table 2:** Patient characteristics.

Criterion	*n*	In-Brace Group (*n* = 25)	*n*	Out-of-Brace Group (*n* = 26)	*p*-Value
Gender, female (%)	25	22 (88.0%)	26	24 (92.3%)	0.61
Age at start of Boston brace treatment	25	11.0 ± 2.7	26	11.7 ± 2.0	0.26
Pre-brace Cobb angle	18	40.0 ± 7.4	26	37.7 ± 7.8	0.32
Pre-brace Lenke classification, curve type 1 (%)	25	20 (80%)	26	24 (92%)	0.20
Brace initiation before menarche (%)	21	15 (71.4%)	24	19 (79.2%)	0.55
Initial in-brace correction	18	19.5% ± 16.4	21	37.3% ± 18.1	<0.01 *
Study follow-up duration (years)	25	3.4± 2.0	26	3.3 ± 1.3	0.78
Duration of brace treatment (years)	25	4.1 ± 2.1	25	3.6 ± 1.6	0.37
Age at surgery	25	15.0 ± 1.6	25	15.3 ± 1.8	0.57
Preoperative Cobb angle out-of-brace †	11	57.9 ± 8.0	26	52.5 ± 9.7	0.11

Values are presented as mean ± standard deviation. Abbreviations: *n*, number of patients for which criterion could be determined; IS, idiopathic scoliosis. * indicates a statistically significant difference (*p* < 0.05). † measured on the last out-of-brace radiograph before surgery.

**Table 3:** Curve progression over time.

Measurement	Estimate in CA In-Brace Group	Estimate in CA Out-of-Brace Group	Mean Difference	SE∆	*p*-Value	95% CI∆
Mean curve progression ^1^	22.9 (SD = 15.3)	15.2 (SD = 7.9)	7.6	3.4	0.03 *	0.80–14.45
Monthly curve progression ^2^	0.5	0.5	0.0	0.1	0.80	−0.09–0.11

^1^ Curve progression was calculated by subtracting patient’s baseline CA from CA at end of brace treatment. An independent t-test was used to test for differences in the degree of curve progression between both groups. ^2^ The estimated monthly Cobb angle progression was based on the mixed-effect model. * indicates a statistically significant difference (*p* < 0.05). Measurements are expressed in Cobb angle degrees. Abbreviations: CA, Cobb angle; SD, standard deviation; SE**∆**, standard error of difference; CI**∆**, confidence interval of difference.

## Data Availability

The authors confirm that the data supporting the findings of this study are available within the article. Raw data were generated at UMC Groningen. The data that support the findings of this study are available on reasonable request from the corresponding author.
